# Unveiling the mechanisms and challenges of cancer drug resistance

**DOI:** 10.1186/s12964-023-01302-1

**Published:** 2024-02-12

**Authors:** Sameer Ullah Khan, Kaneez Fatima, Shariqa Aisha, Fayaz Malik

**Affiliations:** 1 Division of Genitourinary Medical Oncology, The University of Texas MD Anderson Cancer Center, Holcombe Blvd, Houston, TX 77030 USA; 2 Division of Cancer Pharmacology, CSIR-Indian Institute of Integrative Medicine, Srinagar-190005, Jammu and Kashmir, India; 3 Academy of Scientific and Innovative Research (AcSIR), Ghaziabad-201002, India

**Keywords:** Autophagy, Apoptosis, Drug resistance, Cancer stem cells, Multi-drug resistance, Immune cells

## Abstract

Cancer treatment faces many hurdles and resistance is one among them. Anti-cancer treatment strategies are evolving due to innate and acquired resistance capacity, governed by genetic, epigenetic, proteomic, metabolic, or microenvironmental cues that ultimately enable selected cancer cells to survive and progress under unfavorable conditions. Although the mechanism of drug resistance is being widely studied to generate new target-based drugs with better potency than existing ones. However, due to the broader flexibility in acquired drug resistance, advanced therapeutic options with better efficacy need to be explored. Combination therapy is an alternative with a better success rate though the risk of amplified side effects is commonplace. Moreover, recent groundbreaking precision immune therapy is one of the ways to overcome drug resistance and has revolutionized anticancer therapy to a greater extent with the only limitation of being individual-specific and needs further attention. This review will focus on the challenges and strategies opted by cancer cells to withstand the current therapies at the molecular level and also highlights the emerging therapeutic options -like immunological, and stem cell-based options that may prove to have better potential to challenge the existing problem of therapy resistance.

Video Abstract

**Supplementary Information:**

The online version contains supplementary material available at 10.1186/s12964-023-01302-1.

## Introduction

Presently one of the challenging aspects of anti-cancer treatment is drug resistance where cancer cells become forbearing to the treatment thus worsening the conditions of patients [1, 2]. Although, various cancer types are initially sensitive to pharmacological agents, over the time they acquire resistance and attain more aggressive nature [3, 4]. Advances made in discovering targeted therapies in recent years led to the approval of various impactful anti-cancer agents, nonetheless, resistance still owes a big hindrance to their success besides accounting for their life-threatening side effects [5, 6]. Cancer cells show evolving behavior of recurrence, dormancy, and drug resistance even after using conventional treatments (surgery, chemotherapy, etc.) mostly contributed by vicious cancer stem cells (CSCs) [7, 8]. Advanced and more potent chemotherapeutic drugs have been able to succeed the previously available anticancer drugs individually or chronologically or in combination with prevailing treatments [9]. Moreover altered chemotherapeutic dose intensity tactics like intermittent administration or higher doses along with supplements and growth factors to suppress the side effects on bone marrow have proved to be effective in preventing the regrowth of tumor [10–12]. Regardless of this, cancer drug resistance remains to be a major hurdle in medical oncology, therefore understanding the resistance mechanisms (innate as well as acquired) and developing next-generation targeted therapies is crucial for medical need [13, 14].

A complex interplay between intrinsic (innate) and extrinsic (acquired) factors of the cancer cell contributes to cancer resistance towards various therapies. Intrinsic factors include pre-existing genetic mutations, tumor heterogeneity, and activation of intracellular defense pathways, that confer resistance by activating various oncogenic pathways, altering drug targets, desensitization towards therapies, enhancing DNA repair mechanisms, as well as activation of survival pathways, thereby potentiating cancer cells to evade the cytotoxic effects of treatments [15]. While extrinsic factors mainly include tumor microenvironment (TME) components that actively participate in cancer cell ability to evade the cytotoxic effects of various anticancer therapeutics[16, 17]. The various TME components include the altered extracellular matrix (ECM), tumor-associated stromal cells, growth factors, extracellular vesicles (EVs), immune cells, etc. Tumor-associated rigid and condensed ECM affects the drug response by reducing drug transport and sequestering drugs through direct binding with it thus represents a significant mechanism of drug resistance in many solid tumors [18, 19]. Cancer-associated fibroblasts (CAFs) are another TME component that plays significant roles in tumor growth, metastasis and cancer therapy resistance by secreting various growth factors like Hepatocyte growth factor (HGF) and Epidermal growth factor (EGF); cytokines such as stromal cell-derived factor 1 (SDF-1) and interleukin-6 (IL-6) [20, 21]. Extracellular vesicles (EVs) of drug-resistant cancer cells can sequester and transport drugs to ECM. Moreover, EVs may be transferred from drug-resistant cancer cell to drug-sensitive counterparts and thus plays a role in the horizontal transfer of drug resistance in cancer cells by delivering specialized cargo which includes drug resistance-related proteins (P-gp, ABCG2, ABCA3, etc.), nucleic acids (mt DNA, mRNAs, miRNAs), onco-metabolites, antiapoptotic proteins [22, 23]. Targeting drug-resistant cancer cell-intrinsic and tumor microenvironment components, alone or in combination with anticancer therapies may prove to be a better approach in enhancing the efficacy of cancer treatments and improving patient outcomes. The details mechanism of the extrinsic and intrinsic factors implicated in drug resistance and the strategies to inhibit them are discussed in the below sections.

**Fig. 1:**
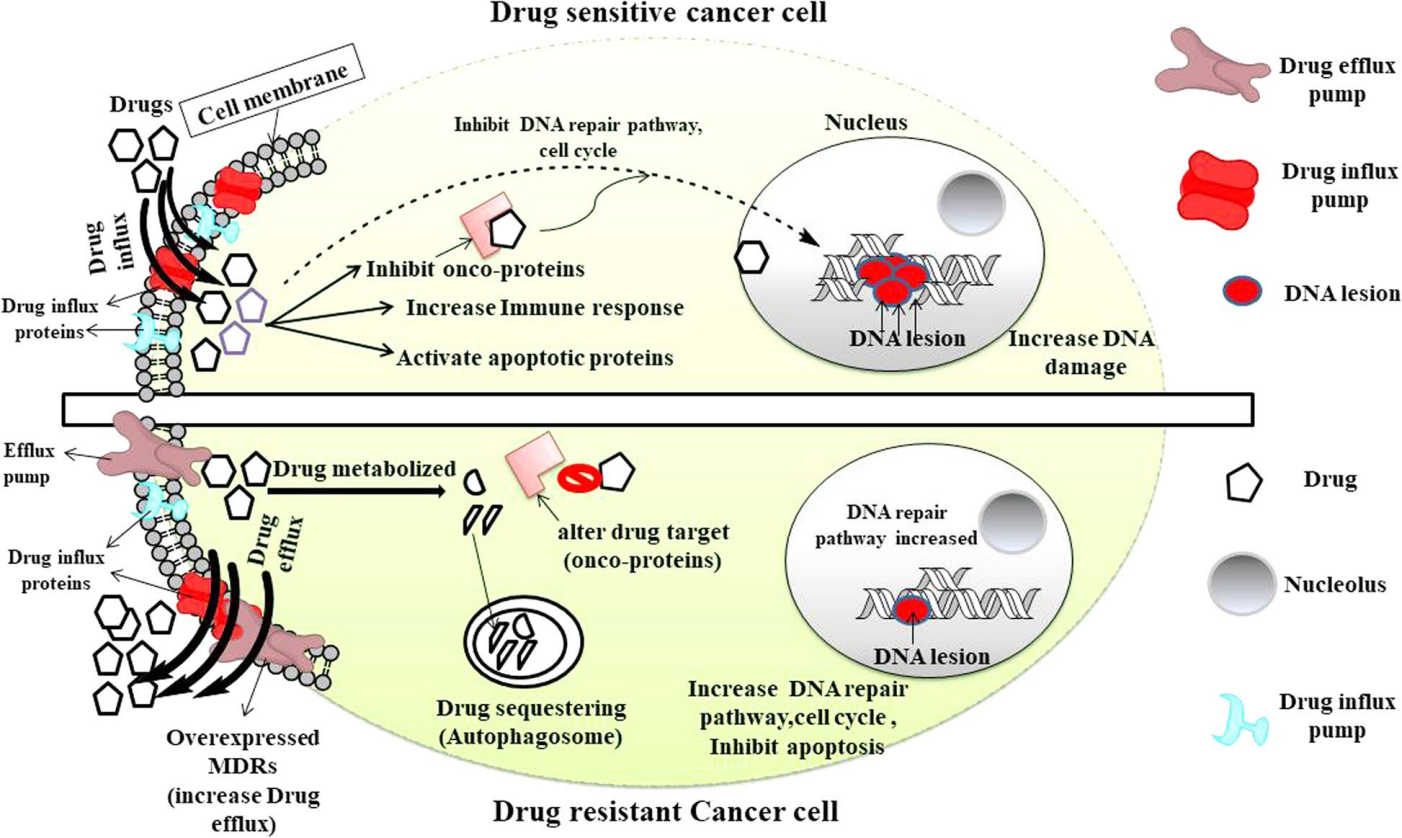
Cancer cell and drug-resistant cancer cell response to an anti-cancer drug. Chemotherapeutic drugs are effective on cancer cells as they enter into the cells and activate various anti-cancer pathways, leading to DNA damage and cell death. In resistant cells, cancer cells modulate drugs or produce a barrier to them which collectively resulted in decreasing their effectivity on cancer cells. Figure created with ChemBioDraw Ultra 14.0

With the advancement in the study of drug-resistance, massive efforts on the development of successful therapies against various factors including RTKs, androgen, HER2 receptors and so on has lead to improved therapeutic options to a greater extent [24–31]. However progressive approaches of using precision immunological therapies were proven to be more successful in the recognition and destruction of cancer cells with more tolerability and better remission. Widely used Anti-CTLA and anti-PD-1/PD-L1 therapy remarkably show antitumor activity by dysfunctioning the negative regulators of the anticancer adaptive immune system, though the minimum chance of resistance and its limitations to a certain subset of cancer remains the concern [32–36]. Cancer cells follow the Darwinian selection pressure rule to achieve drug-resistant traits at genomic, epigenomic and proteomic levels for the survival of their fittest [37–39]. With the advent of high throughput assays, the link between tumor heterogeneity and drug resistance came into existence which suggests that under selective drug pressure, few tumor cells divide and form a subpopulation of cells that may achieve features that enable them to become non-responsive to a particular drug over time [40–43]. Contrasting features of cancer cells and drug-resistant cells are represented in Fig. 1.

In this review, we will discuss the spectrum of selective mechanisms displayed by cancerous cells to resist treatment, which is clinically the more difficult problem. We attempted to interconnect the multidrug-resistant pathways in various cancer types and acquire insights into these difficult aspects to support the development of next-generation cancer therapies such as more potent medicines and immuno therapies. Furthermore, this review tries to identify common themes and approaches that have been used successfully to target emerging resistance mechanisms.

## Tumor heterogeneity is a decisive factor for drug resistance

The biology of normal cell transformation into cancer cells has been explained at the genetic/epigenetic and proteomic levels. However, reasons (usually random) for cancer initiation, development, and progression are not fixed and rather can be considered as unified perplexing dysregulation of crucial cellular processes [44, 45]. Cancer cells are always under stress and are continuously evolving, trying to adjust to the changing environment resulting in the emergence of a heterogenous population of cancer cells in the tumor that differ from each other at the molecular level. Moreover, their level of response to anti-cancer drugs also varies to a greater extent. Tumor heterogeneity is categorized into either intertumoral (heterogeneity between patients due to varying germline, somatic and environmental factors) or intratumoral (heterogeneity within patients due to uneven distribution of genetically diverse tumor subpopulations). The advent, advancement, and access to the genomic landscape (particularly, oncogenic drivers) of aggressive cancers like non-small-cell lung cancer (NSCLC), has updated the clinical strategies towards the personalized or genotype-channeled approach with better outcomes [46, 47]. Despite the initial positive response, cancer cells develop resistance to targeted therapy in the long run of treatment indicating that cancer is very dynamic at the molecular level. Various studies have shown that intratumoral heterogeneity plays a crucial role in driving the advancement of cancer and drug resistance [48, 49]. Numerous factors are responsible for intratumoral heterogeneity, the most important is genomic variability caused by mutagen exposure like UV radiations, chemicals, chemotherapy, or dysregulation of signaling processes that maintain DNA repair, and redox balance of cells [42, 50–53]. The advanced high-throughput assays of large-scale genome sequencing enable us to highlight various genetic signatures associated with genetic instability and variability. For example, lung cancer caused by smoking was shown to be enriched with C → A transversion at the genomic level [54, 55]. Different studies have shown that cancer progression is dependent on the abrupt mutation rate in certain cell types which upsets the balance between oncogenes and tumor suppressor genes thus promoting genetic diversity [56–58]. Another factor that participates in tumor heterogeneity is clonal evolution which follows the Darwinian selection hypothesis [59–61]. According to this hypothesis, continuous division and chromosomal instability lead to a random chromosomal loss in various tumor regions that result in mutational heterogeneity with the outcome of the rise of evolving competitive sub-clones and Cancer stem cells (CSCs) and vice versa [62–64]. These clones formed so far expand either sequentially or by a branched approach and generate more genetic diversity in either way which is further selected under evolutionary pressure with better growth, resistance, and survival advantage [65]. Recent high throughput assays exercises like single-cell RNA sequencing and mutation characterization enable us to investigate and identify the evolutionary dynamics that occur in the particular tumor cell population in the same or different patients and have a flagging role in individualized therapy [66–69]. These diverse genomic changes contribute to acquiring beneficial properties like drug resistance and tumor recurrence in these selected cancer cells [68]. Tumor evolution contributes to the emergence of a multi-drug resistant (MDR) subpopulation of cells with varying treatment responses to the drugs than that of the primary tumor cells [70]. Chemotherapeutic pressure on the other hand plays an important role in the formation of more evolved resistant sub-clones with poorer outcomes [49, 70]. Recent studies have shown that co-existing tumor heterogeneity and immune landscape analysis in a cohort of patients with HBV-associated human hepatocellular carcinoma (HCC) inhibits the T-cell infiltration and thus regulates the intratumoral immune suppressive microenvironment [71]. The finding is crucial to design effectual immune therapies that can be given individually or in combination with existing chemotherapies to sensitize the resistant cancer cells with better outcomes. Thus tumor heterogeneity and varied immune landscape are big hurdles to understanding the resistance and more emphasis on this difficult issue can give a better future direction to the cancer therapy.

## Genetic and epigenetic alteration as an adaptive response to chemotherapy that majorly influences the drug resistance

The study of hereditary phenotypic variations without change in the DNA sequence is referred to as "epigenetics" and was initially introduced by Conrad Waddington (1940), who linked this term with gene and gene products [72]. Epigenetic variations are mostly caused by modifications in gene expression and function by histone modifications (acetylation, phosphorylation, ubiquitination, sumoylation, methylation) and DNA methylation [73]. Numerous investigations have demonstrated the importance of epigenetic modifications in drug-tolerant persister (DTP) cells and their role in the increased tolerance to higher drug pressure [74, 75]. Additionally, it has also been suggested that altering the epigenetic landscapes by DNA methylation (histone/non-histone changes) aids in the maintenance and survival of CSCs which have been shown to exhibit resistance features at the individual level [76]. So focusing on the epigenetic changes concerning the acquired resistant feature can be significant to find more potent targets with better efficacy. Numerous cancer hallmarks, including cellular proliferation, infiltration, metastases, and therapy response, have been linked to specific patterns of DNA methylation (DNAm) [77, 78]. Studies have shown that hyper and hypo-DNAm by DNMTs (DNA Methyltransferases) result in transcriptional silence of tumor-suppressive genes and transcriptional activation of proto-oncogenes respectively [78]. Further, it has been shown that hyper-methylation of the miR-129-5p CpG island induces miR-129-5p suppression, promoting chemo-resistance in gastric carcinoma cells [79]. The 5-azacytidine (5-AzaC; DNA methyl transferase inhibitor) treatment significantly decreased the chemo-resistance to cisplatin, 5-FU, and vincristine in gastric carcinoma MDR cell line SGC7901/VCR via restoring the activity of miR-129-5p by reducing their gene methylation status [79]. Hypo-methylation at promoter regions of various genes may likewise be used by tumors to develop resistance to chemotherapy. For example, it was found in the MCF-7 cell line, the promoter region of GSTp, MDR1,uPA , and O(6)-methylguanine DNA methyltransferase (MGMT) are significantly methylated but in drug-resistant MCF-7 cells, these promoters were hypo-methylated and had a significant role in resistance. MDR1 gene’s hypo-methylation was linked to drug efflux protein (P-glycoprotein; P-gp) overexpression which in turn is responsible for resistance to doxorubicin (DOX) [80–83].

Furthermore, the level of DNA methylation is also crucial in regulating the fate of cells in many malignancies. At the transcriptional and post-translational levels, various anti- and pro-apoptotic signaling components are epigenetically controlled that affects apoptosis sensitivity or resistance. Apoptosis resistance develops when anti-apoptotic proteins like Bcl-2, Bcl-xl, IAPs, etc., and pro-apoptotic molecules like Bid, Bax, Bim, PUMA, and Noxa are expressed or activated oppositely respectively [84]. Baharudin and coworkers (2017) conducted DNAm sequencing on 5 recurrent and 43 non-recurrent colorectal carcinomas (CRC) patients undergoing 5-fluorouracil (5-FU) chemotherapy [85]. They found that the recurrent CRC group showed 4,787 differential methylated genes with 3,112 hypermethylated and 1,675 hypomethylated compared to the non-recurrence group. The hypermethylated genes were linked to the MAPK signaling pathway which is involved in the regulation of apoptosis and therapeutic resistance towards 5-FU in them [85]. The study also found that the administration of 5- AzaC administration improved 5-FU responsiveness in CRC SW480 cell lines [85]. These findings revealed that DNA methylation plays an important role in the development of therapeutic resistance, and targeting it in carcinoma patients would be an open option for a therapeutic approach. Histones are DNA-binding proteins and their binding affinity decides the DNA transcription. The binding affinity of Histone is dependent on epigenetic modifications of lysine or arginine residue catalyzed by histone lysine methyltransferase (KMTs) and protein arginine methyl transferase (PRMTs) both having a strong role in therapeutic resistance [86]. The most frequent KMT is G9a, which catalyzes the H3K9me1/2methylation a reversible silencing gene modification. Liu and coworkers ( 2017) showed a correlation between G9a expression in head and neck carcinoma patients with the anti-cancer drug response. The immunohistochemical analysis further revealed that patients overexpressing G9a were less sensitive to cisplatin than patients with lower expression of G9a [87]. Moreover, G9a has been shown to activate the GCLC (glutamate-cysteine ligase catalytic subunit) which increases cellular antioxidant glutathione (GSH), which protects against DNA damage by cisplatin and thus supports therapeutic resistance [53, 87]. Likewise, PRMTs-mediated modifications are also shown to be responsible for drug resistance in ovarian carcinoma towards cisplatin [88]. Chromatin and associated proteins were most affected by the PRMT1 modification, which resulted in genotoxic stress. It has been established that PRMT1 is recruited by DNA-PK (DNA-dependent protein kinase) to chromatin where it catalyzes the methylation of H4R3 and contributes to the stimulation of genes involved in the senescence-associated secretory phenotype (SASP), in turn increases tumor cells' resistance to cisplatin by shielding them from DNA damage [88].

## CSCs play a key role in developing drug resistance and tumor relapse

Cancer stem cells (CSCs) are evolving cells that can self-renew and differentiate into other types of cancer cells. CSCs are identified by their surface markers as CD34^+^/CD38^-^ in leukemia, CD44^+^/CD24^-^ in breast cancer, etc. John Dick (1997) was the first to isolate leukemia stem cells in acute myeloid leukemia (AML) patients [89, 90]. CSCs are known to play a role in tumor heterogeneity, metastasis, resistance, dormancy, and tumor relapse [21, 91–93]. Numerous pathways are involved in CSCs' self-renewal as well as chemoresistance like Notch, Wnt, TGF-β, and Hedgehog, targeting them has proven to be a promising therapeutic approach to overcome resistance. Various reports demonstrated that the expression of Notch1 plays a central role in increasing trastuzumab resistance in BT474, SK-BR3, and MCF-7 cells, and its inhibition (genetic or pharmacological) sensitizes these cells to trastuzumab [94–96]. Recently, Wang et al. (2022) reported that in prostate cancer stem-like cells, inhibition of the Notch-1 pathway by PF-03084014 ( γ-secretase inhibitor) increases the anti-cancer activity of docetaxel by reducing cell growth, sphere formation and inducing apoptosis [97, 98]**.** Furthermore, CSCs exhibit critical features of embryonic stem cells (ESCs) in terms of transcriptional factor expression (SOX2, OCT4, NANOG, MYC, KLF4, SALL4, and FOXM1) and signaling pathways (like Wnt/β-catenin, Hedgehog, Hippo, Notch, and TGF-β) [99–101]. Studies reported that CSCs invariably hijack the pluripotent or oncofetal drivers like OCT4, SOX2, KLF4, MYC, SALL4, FOXM1, Wnt/β-catenin, Hedgehog, Hippo, TGF-β of ESCs [99]. For example, the oncofetal circulating CSCs marker “Lin28B” is associated with the recurrence of hepatocellular carcinoma and acts as an ideal therapeutic target [102]. Targeting oncofetal stem cell markers are epitome therapeutic targets as they are not expressed in normal stem cells and can be exploited in various cancer types with better outcomes [103].

Conventional therapies have been found to give rise to CSCs which later play a role in tumor relapse and therapy resistance as shown by various in-vitro and in-vivo studies [104]. For example, radiation therapy-induced glioblastoma CSCs (CD133^+^ /Prominin-1) formation supports radioresistance by activating DNA checkpoints and repair pathways. Thus glioblastoma radiosensitization was increased with the co-treatment of checkpoint or kinase inhibitors (Chk1 and Chk2) and radiotherapy [105]. Additionally, it was found that using a humanized monoclonal anti-VEGF antibody (Bevacizumab) was initially effective in decreasing tumor formation in Glioblastoma multiform (GBM) [106–108]. But due to the formation of resistant lineage and VEGF-VEGFR2-Neuropilin-1 autocrine signaling dominance over time, the clinical benefit lasted for a short period and later resulted in tumor relapse [107, 109]. Experimental investigations of urothelial bladder cancer (UBC), cisplatin, and gemcitabine-resistant cells (T24 and 5637) showed increased expression of CSCs compared to their chemo-sensitive counterparts via miR34a/GOLPH3 axis [110, 111]. Table 1 highlights the clinical trails targeting CSCs to overcome treatment resistance. Thus accumulating investigations insights us that existing CSCs pose a major hurdle in the currently available treatment strategies to restrict tumor relapse thus pushing our special focus on exploring novel CSCs targeted therapy.

**Table 1:** Clinical trials using novel drugs that target CSCs

Drug Name	Experimental study	Type of Cancer	Clinical Phase	Reference
Sonidegib (LDE225)	LDE225 has the potential to disrupt CSC niches and overcome docetaxel resistance	TNBC	1b	[112]
RO4929097	CSC-mediated antiandrogen resistance, tamoxifen resistance, and radiation resistance are reversed	Recurrent Malignant Glioma	1	[113]
PF-03084014	Counteracting docetaxel resistance in CSC	Desmoid Fibromatosis	1	[114]
PRI-724	It could overcome cisplatin resistance in CSCs and reduce the expression of SOX2 and CD44	Hepatitis C Virus-related Cirrhosis	1	[115]
Vismodegib (GDC-0449)	It has the potential to overcome radiation, carboplatin/erlotinib resistance as well as stemness	Multiple basal-cell carcinomas (MIKIE)	2	[116]

### Dysregulated developmental Cues that regulate CSCs contribution to chemoresistance

Solid link between chemotherapy resistance with CSCs is not well explored but they evade the cytotoxic effect of the drug efficiently. However, some evidence suggests that CSCs amplified epigenetic changes, drug transporters, dormancy, and EMT-MET transitions that have a definite role to play in developing resistance [117, 118]. Dormancy is a key feature of CSCs that assist a small population of cells to survive under cytotoxic treatments that are known to be responsible for tumor relapse in the long run [119]. Additionally, reports suggest that CSCs exhibit higher expression of membrane ABC transporters, which expel drugs out of the cell and thus support resistance and tumor relapse [120]. For example in triple-negative breast cancer (TNBC) CSCs, overexpression of ABCG2 has been linked with chemoresistance [121]. Additionally, Sissung TM et.al (2010) reported that expression of ABCG2 provides resistance to 5-FU, and doxorubicin in various cancer cell types by expelling drugs outside and thus protecting them from apoptosis [122–125]. CSC markers have been reported to promote multi-drug resistance by modulating drug efflux pumps. For example, CSC marker p-CD44 (Ser-291) prevents FBXO21 (Ubiquitin E3-ligase) mediated proteasomal degradation of P-gp on breast and ovarian cancer cells and thus remains active in expelling drugs from the cells and prevents its cytotoxic effects [126]. Moreover, recent high throughput studies have demonstrated that CSCs mostly reside in the region of low pH, fewer nutrients, and the hypoxic niche of tumors which evolve cells to progress in stressful conditions [127]. Hypoxia-mediated gene induction promotes CSC drug resistance by upregulating the expression of various types of ABC transporters such as MRP1 which is a downstream target gene of the HIF-1α axis [128]. Due to poor vasculature, drug distribution to cells residing in the hypoxic region is insufficient that provides them the advantage to survive and evolve with time to withstand the cytotoxic effect of the drug [129]. Moreover, the hypoxia-mediated acidic environment around the tumor acts as a physiological and chemical barrier against certain drugs [130, 131]. The above description provides us insights into how CSCs utilize the different processes and environmental factors to proliferate and survive under unfavorable conditions.

Cell survival and cellular functions under nutrient deprivation, hypoxia, or in drug resistance, depend on an evolutionary conserved physiological process known as autophagy [53, 132–134]. Interestingly cancer and CSCs exploit this catabolic process to support tumorigenesis, maintain pluripotency, tumor progression, and relapse [135, 136]. For example, CD44^+^CD117^+^ ovarian CSCs showed increased basal autophagy compared to their non-stem cell counterpart, thus inhibiting autophagy by CRISPR/Cas9 ATG5 knockout making these CSCs chemosensitive [137]. Autophagy is known to reduce the chemotherapy-mediated oxidative stress in normal, cancer and CSCs thus protecting them from cell death [53, 138, 139]. Similarly, in cancer stem cells and normal stem cells, the marker enzyme aldehyde dehydrogenase (ALDH) oxidizes intracellular aldehydes and shields them from harmful consequences of reactive oxygen species (ROS) [140]. A surprising study has shown that the ALDH isoform (ALDH1A3) is responsible for lower doses of Temozolomide resistance in glioblastoma. Higher doses of Temozolomide were shown to induce direct physical interaction of ALDH1A3 with autophagy adaptor protein p62 thus leading to their degradation and reducing the resistance [141]. Yeo et al. (2016) demonstrated the tumorigenic dependence and stemness of ALDH^+^ and CD29hiCD61^+^ breast cancer stem cells on autophagy which act through EGFR/STAT 3 and TGF-β/Smad signaling respectively [142]. Similar findings in TNBC CSCs showed that stemness (CD44^+^/CD24 Low) maintaining and microenvironment-modulating cytokine IL-6 secretion is regulated by autophagy through JAK/STAT pathway. This highlights the importance of the IL-6-JAK-STAT3 pathway axis in CSCs development thus promoting drug (chemo/immune therapy) resistance in them [143–145]. Thus using IL-6 inhibitors like Tocilizumab (humanized IL-6R antibody) overcomes docetaxel resistance in TNBC CSCs by restricting the autocrine action of IL-1 on IL-6 induction [146].

### CSCs control the host immune system and mediate drug resistance

Usually, traditional therapies kill cancer cells but cannot eliminate the small population of tumor cells known as CSCs or tumor-initiating cells (TIC), though they can be recognized and eliminated by the host immune system to a greater extent. However tumor microenvironment intervene the immune offense of tumor elimination and promotes immune suppression thus shifting equilibrium and later escape of CSCs [147, 148]. In various cancers like glioblastoma, lung, breast, etc., M1 (classically activated or pro-inflammatory) macrophages are attracted by chemotaxis (towards cytokines released by CSCs) to the tumor site where they get converted to M2 (alternatively activated or anti-inflammatory or tumor-associated) macrophages, secreting TGF-*β*, IL-10, IL-23, and arginase 1 that creates immune-suppressive tumor microenvironment for tumor growth [149–152].

CSCs' intrinsic immunosuppressive system releases cytokines such as IL-10, IL-4, IL-6, MIC-1, CCL2, CSF1, CSF2, HGF, MIF, CX3CL1, CSF2, PGE2, SDF-1, Periostin (POSTN), CCL2, LOX, CCL3, CCL5, VEGF-A, NTS exosomes and IL-8 that collectively plays a pivotal role in recruitment, polarization and ultimately M1/M2 macrophage conversation [153, 154]. M2 in turn maintains CSCs features by releasing signaling molecules like CCL2, CCL5, CCL7, CCL8, CCL17, CXCL1, CXCL7, PTN, HMGB1, TGF-*β,* IGF, IL (10, 1β, 6, 8, 18, 35), etc. and thus support resistance and relapse of tumor [154]. For instance, in hepatocellular carcinoma (HCC), CD133^+^ cells promote the M2 polarisation of TAMs by the release of IL-8 which in turn is responsible for the therapeutic resistance [155, 156].

**Fig. 2:**
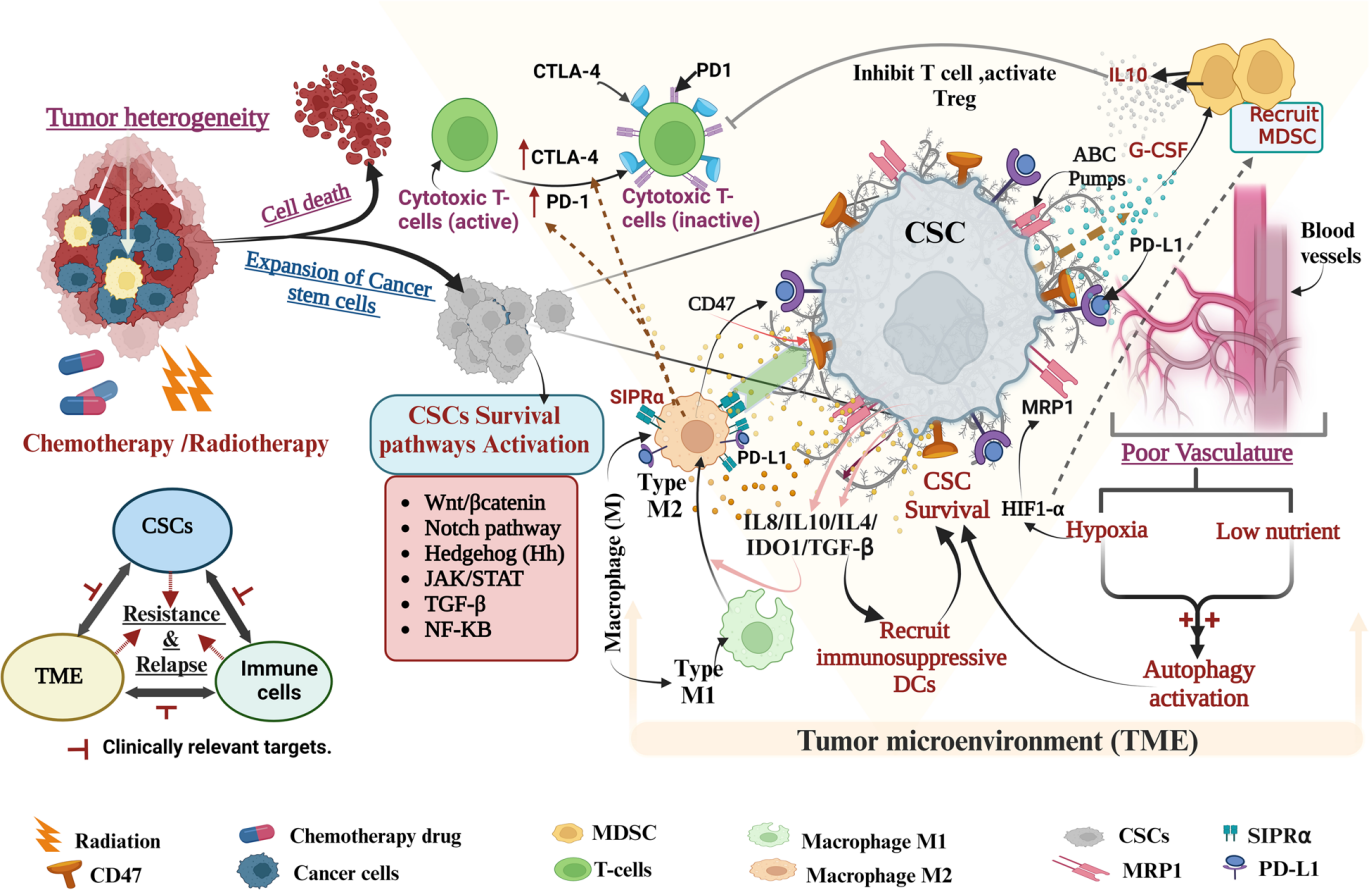
Role of CSC in drug resistance and relapse by altering its microenvironment and modifying the host immune system. CSCs are resistant to various therapies compared to cancer cells by activating various survival pathways and also changing their microenvironment like increasing autophagy as well as secreting various chemokines and cytokines which in turn cause drug resistance. Figure created with BioRender.com

CSCs-activated TAMs also inhibit T-cell cytotoxicity by overexpressing cancer immune checkpoint receptors such as programmed death ligand protein1 (PD-L1), and (CD80/CD86)  that  interact with programmed cell death protein-1 (PD-1) and cytotoxic T-lymphocyte-associated protein-4 (CTLA-4) on the surface of CD8^+^T cells respectively, impairing the immune response and support anti-tumor immune resistance [157–159]. Additionally, HCC CSCs hijack TAM SIPRα-CD47's “Don’t eat me” signaling pathway to evade immune surveillance and prevent them from being phagocytosed by macrophages [160]. Surprisingly, blocking overexpression of the CD47 receptor in gefitinib resistant EGFR mutated NSCLC with a monoclonal antibody improved the phagocyte clearance of these cells [161]. This study supports the use of targeted monoclonal antibodies to neutralize CD47 as a promising immunotherapeutic approach for resistant EGFR-mutant NSCLCs. CSCs can control, antigen-presenting dendritic cells (DC: T & B-cell memory development) tolerance and prevent them from activating T cells [162–165]. Additionally, CSCs produce immunosuppressive cytokines (like IL-4, IL-10, TGF-β, etc.), and co-inhibitory molecules (like IDO1, PD-L1, and B7-H3) that attract immunosuppressive DCs to suppress the anti-tumor immune system and activate/recruit immune suppressing Tregs [159, 166]. In glioblastoma, it was shown that CSCs promote Treg cell infiltration, reduce cytotoxic T-cell activation, and induce T-cell apoptosis by soluble Galectin-3 and B7-H1 signaling molecules [159]. It has been reported that CSC increases the production of G-CSF (Granulocyte colony-stimulating factor) which in turn recruits MDSC (Myeloid-derived suppressor cells) to the tumor site via mTOR signaling pathway and the amount of infiltrating MDSC is positively co-related to CSC existence in cancer patients [156]. MDSC produces immunosuppressive cytokines such as IL 10, TGF-β, etc., increases PD-L1, and prostaglandin E2 (PGE2) expression, and recruits Tregs which collectively suppresses the T-cell (CD8^+^), maintains stemness (like in ovarian CSCs), and drug resistance[167–169], as shown in Fig. 2. In hepatocellular carcinoma, the hypoxia-mediated CSCs attract MDSCs to the tumor site through ENTPD2/CD39 L1 signaling and halt the PD1 treatment, and the reduction of MDSC sensitizes these cells to 5-FU [170, 171]. Recently introduced CAR T cell (chimeric antigen receptor T cell) is an altered host white blood cell therapy, that has revolutionized anti-cancer immune therapy to cross the barrier of specificity and non-responders to standard therapy and was first tried on acute lymphoblastic leukemia (ALL) patients with better remission [172–174]. However, due to the constant threat of developing cytokine release syndrome (CRS) and early relapse of antigen-positive leukemia (loss of active CAR T cell-mediated surveillance) or later relapse (loss of antigen) of the tumor, limited the range of specific CAR T cells [175]. However recent cotreatment of CAR T cells with immunomodulators (immune check point inhibitors like PDL1-PD1 blockage) has shown to be promising in terms of the depth and durability of the treatment clinically [176–179]. The above description insights us into the importance of the ‘CSC-TME-immune’ triangular (Fig. 2) signaling interaction in tumor expansion and therapy resistance and its clinical significance. A comprehensive representation of immune and CSC in drug resistance linkage has been shown in Fig. 2.

## Dynamic EMT programs and drug resistance are mutually linked to each other

Various studies have shown the emerging role of the Epithelial-to-mesenchymal transition (EMT) program in tumor relapse, regulation of CSC phenotype, therapy hindrance, and anoikis resistance thus is clinically a relevant model to be targeted [117, 132, 180]. EMT is a highly conserved complex cellular program that transforms epithelial cells to attain a range of mesenchymal or CSCs features like increased cell mobility and upregulated drug efflux pumps. EMT transition usually happens under the influence of hypoxia, cytokines, or by activation of TGF-β, Notch, hedgehog, and Wnt pathways [181, 182]. A spectrum of reports suggests that EMT signaling pathways act as a driving force in cancer metastasis and drug resistance [132, 183, 184]. Snail, TWIST, ZEB, N-cadherin, and fibronectin are the signatures of EMT each having a definite role in drug resistance. EMT-activator ZEB1 (zinc finger E-box binding homeobox 1) represses the expression of epithelial phenotype-promoting genes like miR-200, and miR-203 thus promoting stemness and subsequently resistance to chemotherapy [185–187]. ZEB1 and miR200 play a double negative feedback loop and repress each other's function. Various groups have exploited this process and increased the miR200 in cancer cells artificially which induced partial chemosensitivity towards drugs [188–190]. So inhibiting ZEB1 at the epigenetic level by using mocetinostat (Class 1 HDAC inhibitor) successfully restores miR200 and sensitizes pancreatic cancer cells towards chemotherapy [191].

Moreover, a cohort of large breast cancer samples has revealed that ZEB1overexpressing samples were less responsive to genotoxic epirubicin treatment. Upon dissecting the mechanism it was found that ZEB is actively activating ataxia-telangiectasia mutated (ATM) kinase at the transcriptional level by promoting the ZEB1/p300/PCAF complex formation which resulted in the activation of homologous recombination DNA repair pathway [192]. EMT and the tumor cells microenvironment (TME) are linked through the FBXW7-ZEB2 axis to promote colorectal CSC formation and chemoresistance [40]**.** Moreover, through the FZD7/Wnt/β-catenin pathway, SOX8 facilitates EMT processes and supports chemo-resistance of Tongue squamous cell carcinoma (TSCC) [193]. Another EMT promoter TWIST1 stimulation by Metadherin (MTDH) resulted in CSC characteristics and drug resistance in MCF-7 cells [194]. Moreover, Mukherjee et al. also demonstrated that in TNBC cells, the SOX2-ABCG2-TWIST1 pathway plays a significant role in regulating tumorigenicity and chemoresistance [195].

## Role of pH gradient across organelles in drug resistance

Subcellular organelles, despite their role in compartmentalizing the sub-organelle components at optimum; have been also identified to play an essential role in drug resistance [196]. After drug administration, the drug usually enters the cells to reach its targets which are mainly present in cellular organelles such as mitochondria, lysosomes, nucleus, ER, GB, and peroxisome or in the cytosol [197]. The functional features of these organelles, like membrane electrochemical gradient, drug transporter expression, protein compartmentalization, and intraluminal pH are uniquely different from one another. Various cancer cells hijack these features for tumor growth, survival and drug resistance.

**Fig. 3:**
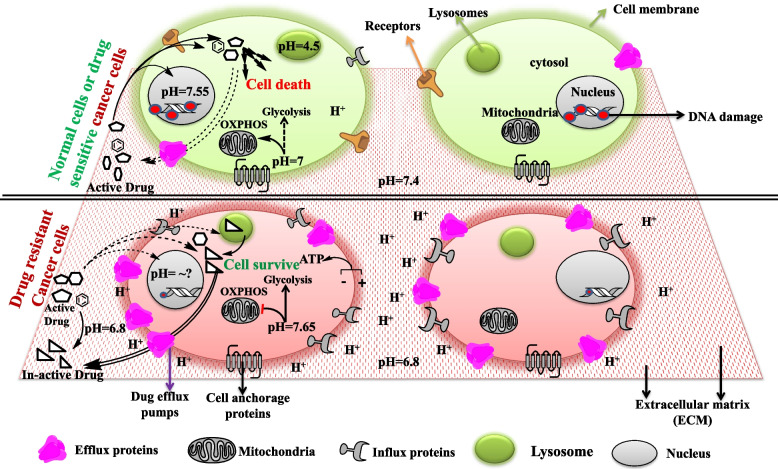
pH change and cellular organelle's role in drug resistance. Resistant Cancer cells change extracellular (ECM) as well as intracellular (cytosol, subcellular organelles) pH according to their requirement thus activating various pathways which cause metabolic, genetic, epigenetic rewiring to support their survival, metastasis, and drug resistance. Figure created with ChemBioDraw Ultra 14.0

The extracellular microenvironmental pH is 7.4 (basic) in normal tissue which is altered in the cancer microenvironment to 6.8 (acidic), favoring the activity of various metalloproteinases, activating several signal transduction pathways and acting as a chemical barrier for many anticancer drugs that exuberate malignancy and tumor aggression [198–203]. Besides it has been found that malignant cells alter their cytosolic pH more towards basic (from 6.99–7.2 to 7.12–7.65) thus generating a proton gradient across the plasma membrane which is utilized for direct generation of ATP [204–206] Fig. 3. Moreover, this altered pH gradient favors aerobic glycolysis instead of OXPHOS in cancer cells for fast energy generation and maintenance of the acidic microenvironment by lactate production which is essential for tumorigenesis and drug resistance [207]. Nuclear pH varies between 7.55–7.88 in normal cells and plays a significant role in DNA-histone and DNA–protein interaction thus influencing nuclear activity like DNA replication, epigenetic modifications, etc. [206, 208]. Since cancer cells proliferate indefinitely and have a higher epigenetic modification rate and thus become venerable to chemotherapy. With less evidence, nuclear pH alteration can act as a hurdle for anti-cancer drug activity and pose resistance to them, thus can be a useful parameter to be exploited to sensitize cells to drugs [209, 210]. The most active pH is found in endolysosomes which varies from 6 in early to 4.5 in late endosomes [211–213]. Oncogenic transformation is shown to change in lysosomal volume and its subcellular location with less effect on pH change [214, 215] Fig. 3. Endolysosomes are an important component of the intracellular catabolic process called autophagy which clears the extra, unknown, deformed, and unused biomolecules from the cells by breaking them into smaller building blocks or energy units for the cell [53, 132–134]. Various reports suggest that endolysosome's pH plays a pivotal role in drug resistance by accumulating and sequestering various chemotherapeutic drugs (a mostly weak base) that enter into them either by passive diffusion or through membrane-embedded P-gp pumps and are later expelled by exocytosis and thus generate chemoresistant cancer cells [216–219]. So the integrity of lysosomal membrane permeability (LMP) is necessary for cancer cells to be resistant and can be considered a therapeutically important subject. Various studies have shown that LMP inducers like chloroquine (CQ) can result in resistant cancer cell death by releasing sequestered drugs and proteasomes like cathepsin to act on the nucleus and induce apoptosis [220–222]. Moreover, CQ promotes the release of NO that efficiently inhibits P-gp activity and leads to the accumulation of chemotherapeutic drugs thus leading to death in the resistant hepatic carcinoma [223].

Mitochondria is another important organelle that generates ROS and ATP for the cell and plays a role in chemoresistance. A recent study has shown that cancer cells use mitochondrial ATP for ABC transporters activity which expels drugs out of cancer cells and is negatively regulated by methylation-controlled J protein (MCJ; endogenous negative regulator of ETC) [224–226]. In MDR NCI/ADR-RES ovarian and doxorubicin-resistant MCF-7 cells, MCJ deficiency was found to be responsible for maintaining the drug efflux pumps and supporting the resistance in them thus highlighting MCJ as a therapeutic candidate [227, 228]. Elisa et al. demonstrated that overexpression of UCP2 (uncoupler protein 2) in pancreatic cancer cells significantly decreases drug-mediated mitochondrial superoxide generation thus protecting them from apoptosis [229].

**Fig. 4:**
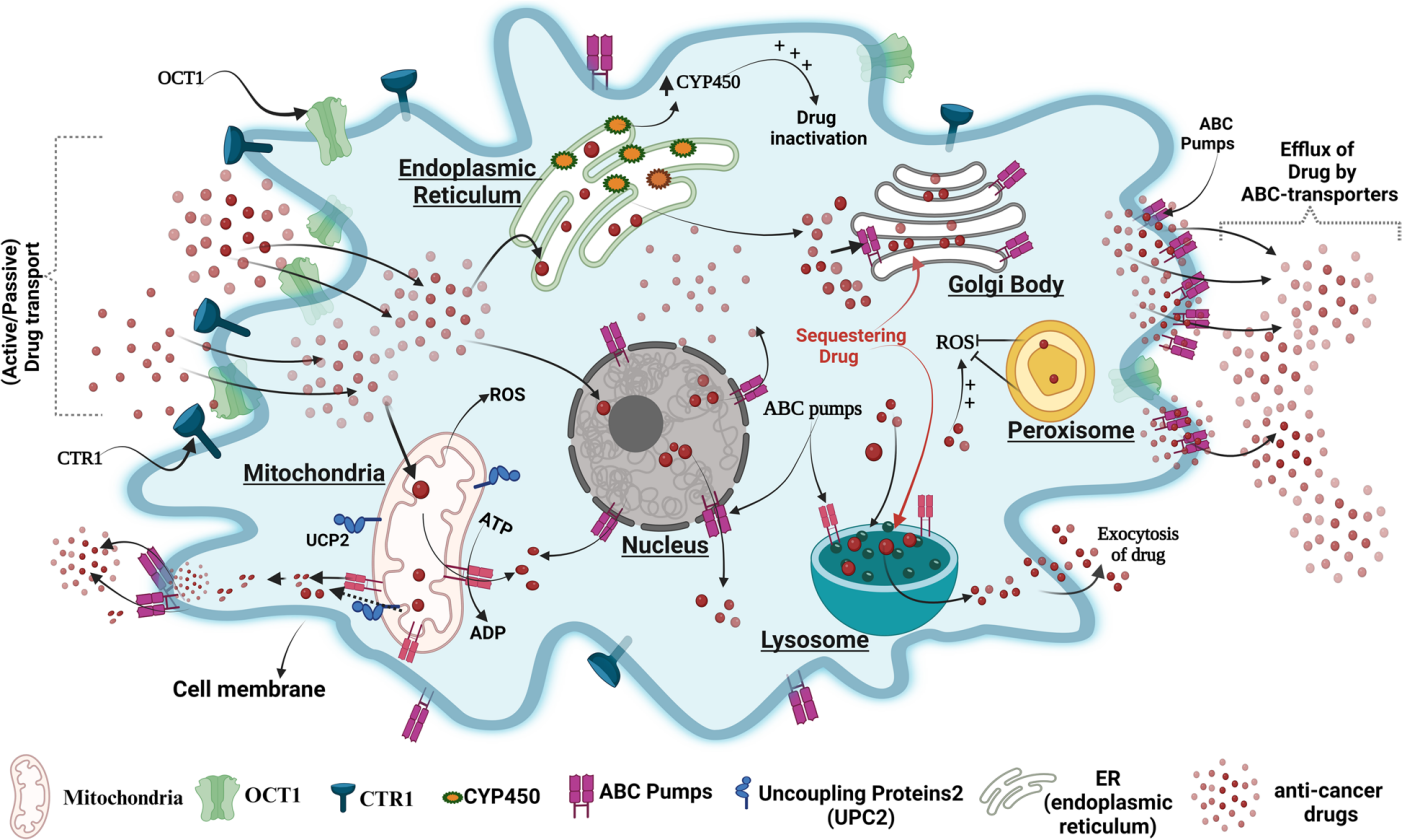
Cancer cells hijack cellular organelles for their benefit. Cancer cells respond to drugs by decreasing ROS production( by peroxisomes), increasing drug efflux pumps(on the membrane of organelles and cells), drug-metabolizing enzyme(ER-CYP) and sequestering drugs (by lysosome and Golgi). Figure created with BioRender.com

Studies show thatEndoplasmic reticulum (ER) plays a vital role in drug metabolism due to the presence of the drug-metabolizing enzyme “cytochrome P450”(CYPs). Lin. et al. demonstrated that the expression of cytochrome P450 enzyme CYP1B1 was higher in taxol-resistant A549 cells compared to its parental A549 cells and inhibition of CYP1B1 by 4 hydroxy-emodin increased their sensitivity to Taxol [230]. The Golgi body (GB) functions as a post-translational trafficking hub and has a role in drug resistance as well. In glioblastoma resistance cells, GB overexpresses various MDRs and sequesters the drugs by their secretary system which later exports drugs out of the cells, the process is reversed by P-gp inhibitors such as S9788 and PSC833, which reverses drug resistance [196]. The peroxisome an oxidative stress reliever of cells also plays a role in resistance as in lymphoma towards vorinostat by reducing ROS generation and inhibition of peroxisome activity was shown to increase the drug sensitivity of these cells [231–233]. The nucleus is the control center of eukaryotic cells and previous studies reported the expression of various kinds of ABC transporters such as P-gp on the membrane of the nucleus and its role in resistance to various anti-cancer drugs like doxorubicin in various cancers such as glioblastoma multiform (LN-299) [234–237]. An image representation of the organelle's role in drug resistance is shown in Fig. 4.

Despite the importance of organelles in cellular function is indispensable, however, different cancer cells hijack their function by molecular rewiring and exploiting them for their survival and drug resistance and thus can be considered as an extra therapeutic edge in the future.

## Immune cells and tumor microenvironment protect cancer cells from anti-cancer drugs

The tumor microenvironment (TME) is a complicated and evolving "rogue organ" made up of stroma, extracellular matrix, immune cells, nervous and lymphatic systems, as well as its blood supply [238]. Cellular crosstalk between cancer cells and the ECM is a significant component of the TME which affects immune evasion and ECM remodeling that promotes tumor initiation, metastasis, and treatment resistance [238–240]. Each microenvironmental component, including cancer-associated fibroblasts (CAFs), immune cells, ECM components, exosomes, hypoxia, and acidic environment, plays a role in concert and contributes to the therapeutic resistance as described below.

**Fig. 5:**
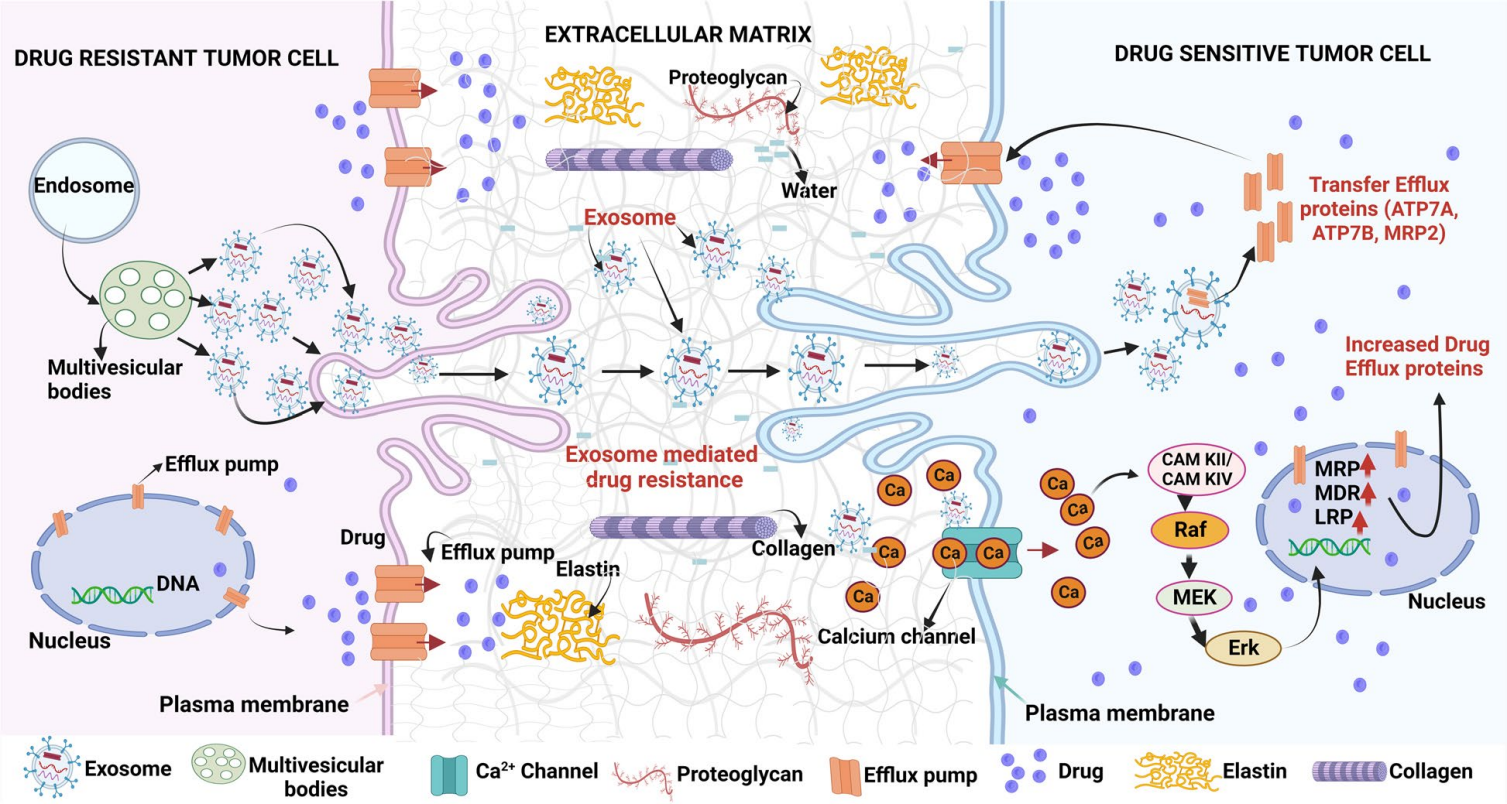
Exosome-mediated drug resistance: Drug-resistant tumor cells can connect with drug-sensitive tumor cells through the exchange of exosomes. Exosomes can transport proteins (such as drug-efflux pumps) and other critical components involved in drug resistance. Mesenchymal stem cells (MSC)-derived exosomes stimulate calcium-dependent protein kinases and Ras/Raf/MEK/ERK kinase pathways which in turn activate multiple drug efflux pumps. Figure created with BioRender.com

i) *CAFs (stromal cells)*: are activated fibroblasts, abundantly associated with cancerous cells in the TME, a variety of oncogenic cues like growth factors, chemokines, exosomes, etc. contributed by CAFs allow cancer cells to undergo EMT, evade therapies and potentiate them for tumor relapse [241–244]. CAF responds to the chemotherapeutic drug cisplatin used against esophageal squamous cell carcinoma (ESCC), by secreting paracrine signaling molecule plasminogen activator inhibitor 1 (PAI-1). PAI-1 in turn stimulates the survival of  AKT and MAPK pathways to protect ESCC from ROS-mediated DNA damage and cell death under chemotherapeutic drug treatment thus supporting resistance [245]. Moreover, CAF-secreted hepatocyte growth factor (HGF) or TGF-β1- stimulates downstream PI3K-AKT/MAPK/ERK/STAT pathways, thus leading to anti-EGFR, anti-BRAF chemoresistance in colon cancer, glioblastoma, melanoma, etc. [246–249].
ii) ECM: The ECM is another factor that is made up of fibrous proteins like collagen, elastin, proteoglycans, microelements, water, etc., and plays an active role in therapeutic resistance (Fig. 5) [250, 251]. Tumor ECM is substantially different in structure and composition and exhibits fibroblastic/myofibroblastic infiltration, followed by the considerable buildup of collagenous matrix or desmoplastic stroma, obstructing anti-cancer drug delivery to cancer cells [251, 252]. It was found that cisplatin-resistant ovarian malignancy overexpresses COL11A1 (collagen type XI α1) which increases the chemoresistance by activating tumor-favoring AKT /PDK1 pathways [253, 254]. ECM drug-resistant signals are transmitted mostly through membrane-bound cellular receptors (like integrins) that lead to significant intracellular rewiring and thereafter evolve cancer cells for therapeutic resistance [255, 256]. It has been shown that cancer cells TME/ECM lacking collagen or fibronectin or both were more sensitive towards cisplatin than their counterparts [257]. In continuing with this, Oxaliplatin's effectiveness against colon cancer cells was increased by suppressing the αv subunit of integrin [258].
iii) Exosomes: Exosomes, also known as extracellular vesicles (EVs), which are produced by big multivesicular bodies (MVBs), facilitate cell-to-cell interaction by transporting bioactive cargos, across cells and efflux of undesirable molecules in healthy cells [259]. They are important signaling intercessors, playing roles in tumor growth, TME remodeling, metastasis, angiogenesis, as well as treatment resistance [260, 261]. Various cancerous cells hijack EVs for anti-cancer drug efflux which in turn results in drug resistance as shown in Fig. 5 [262, 263]. Mesenchymal stem cell (MSC) derived exosomes integrate into gastric tumor cells, enhancing CaM-Ks and downstream Raf/MEK/ERK signaling cascades pathway activation, which increases the expression of MDR-related proteins resulting in gastric carcinoma chemoresistance [264]. Ovarian cancer drug-resistant cells showed increased cisplatin exosomal export along with the recruitment of efflux transporters ATP7A, ATP7B, and MRP2 (ABCB2) in neighboring cells to protect drug-mediated cell death [265].
iv) Hypoxia: The tumor’s aberrant vasculature and high oxygen demand result in hypoxia, and reduced availability of nutrients like glucose and vital amino acids [266, 267]. Oxygen deprivation stimulates hypoxia-inducible factor (HIF)-1α, which regulates many cell survival and angiogenic genes, that in turn favors cancer cells to withstand the cytotoxic effect of chemotherapeutic drugs [268, 269]. HIF-1α promotes survival by two-way processes of either suppressing pro-apoptotic proteins (TRAIL) or stimulating anti-apoptotic proteins, (like c-myc, etc.), under chemotherapeutic pressure like in temozolomide resistance in GBM [270–272].
v) Immune cells: The most prevalent immune cell types in TME are tumor-associated macrophages (TAMs) [273]. TAMs invasion into TME has been linked to poor prognosis and inadequate response to chemotherapeutic agents in cancer patients [274, 275]. TAMs are derived from circulatory Ly6C^+^CCR2^+^ monocytes. TAMs have a high degree of variability in TME and can be classified into two subgroups: (1). classically stimulated pro-inflammatory M1 macrophages exhibiting anticancer characteristics and (2). alternatively stimulated anti-inflammatory M2 macrophages having tumor-supporting capabilities [276]. The M2 phenotype plays a role in treatment resistance and induces Th2 responses. In the cancer microenvironment, the polarization of macrophages from M1 to M2 is prevalent [277]. Drug treatment stimulates TAMs to develop into immunosuppressive M2-polarized macrophages which confer chemoresistance in various cancerous cells. It has been shown that ROS builds up in the gastric carcinoma cells after exposure to 5-FU activates HIF-1α-(HMGB1) signaling, which recruits M2 TAMs, which generates GDF15 (growth differentiation factor 15), and enhances fatty acid β-oxidation thus increasing the chemoresistance in them [278]. To shield cancer cells from therapeutic action, TAMs also secrete a variety of soluble factors into TME, such as interleukins, chemokines, etc. High infiltrations of tumor-associated neutrophils (TANs) within the TME have also been responsible for tumor growth as well as drug resistance [279–281]. T-regulatory cells (Tregs) are a type of immunosuppressive T-cells that are CD4^+^CD25^+^ and are distinguished by the Foxp3 expression that is required for Treg formation and differentiation. Elevated Treg invasion in the TME had been linked to worse prognosis as well as chemoresistance in glioblastoma, melanoma, colorectal, and renal cancer [282–284]. It has been shown that 5-FU therapy increased the expression of chemokine (CCL20) in colorectal carcinoma cells (CRC) in vivo by triggering FOXO1/CEBPB/NF-κB signaling, which aided in the migration of Tregs into TME. Foxp3^+^ expression on Tregs is in turn linked to resistance-related genes such as ATP8A2, BCL2, VIM, and WNT1 which promote chemoresistance to 5-FU in CRC [283].

## Metabolic changes/regulations govern drug resistance in cancer cells

One of the features that distinguish cancer cells from normal cells is metabolic reprogramming. Cancer cells have more efficient anabolic pathways and the capacity to utilize carbon sources other than glucose [285, 286]. Changes in cellular metabolism not only assist in the development of tumors but also play a role in the resistance of cancer cells to antitumor therapies including resetting lipid metabolism, glycolysis, polyamine synthesis, and so on [287].

As already discussed above due to pH differences cancer cells sustain their high glycolytic rates for rapid ATP production to meet the high energy-demanding processes like activation of glucose transporters (GLUTs) etc. [288, 289]. Active aerobic glycolysis produces lactate as an end product which is expelled to ECM by Monocarboxylate transporter 4 (MCT4) forming an acidic microenvironment that is necessary for tumor growth, metastasis, immunosuppression, vascularization, and therapeutic resistance [290]. Moreover, lapatinib-resistant BT-474 breast cancer cells have shown to be dependent on enhanced glycolytic enzyme (Aldolase A) activity which reversibly forms glyceraldehyde 3-phosphate from fructose-1,6-bisphosphate, thus are susceptible to glycolysis inhibition [291]. Further reports suggest the resistance dependence of cancer cells on glycolytic intermediate and glucose transporter are therapeutically significant nodes in both preclinical and clinical settings [292]. In Gemcitabine-resistant pancreatic cells, glycolysis inhibition by using a 2-deoxy-D-glucose (2-DG) increased the cytotoxic effect of the drug by suppressing CSC phenotypes in both in-vitro and in vivo [293]. In the stomach, ovarian, breast, hepatocellular, and cervix malignancies, one of the key glycolytic enzymes Hexokinase-2 (HK2) is shown to increase drug (cisplatin) resistance and support their survival [294]. In breast cancer, HK2 binds to a mitochondrial voltage-gated ion channel (VDAC) and blocks drug-induced intrinsic apoptosis [295]. The above evidence highlights that VDAC or glycolytic inhibitors are concomitant candidates for anti-cancer therapy and co-targeting them could prove very effective clinically.

Moreover, ATP metabolism also has a profound role in the onco-immune or immunomodulatory system which in normal conditions maintains the body's homeostasis by maintaining the balance between immune-stimulatory, immune suppression, and autoimmune disease defense. Cancer cells metabolize extracellular ATP into immunosuppressive extracellular adenosine (eADO) by transmembrane ectopeptidases CD39 (ectonucleotidase triphosphate diphosphate-hydrolase 1) and membrane-anchored CD73 (5’-nucleotidase) [296]. Although another noncanonical pathway also participates in the generation of adenosine which includes the activity of CD38 (NAD^+^ ectohydolase), CD203a (ectonucleotidase pyrophosphatase/phosphodiesterase family member1), tissue-specific (for example prostatic acid phosphatase) and tissue-nonspecific alkaline phosphatase (TNAPs) collectively making the TME immune suppressive [297]. Adenosine acts as a ligand for adenosine-specific receptors that are widely distributed in all types of cells including immune cells. Adenosine receptors (P1 type G-protein coupled receptors) are of four distinct types A1, A2A, A2B, and A3, and work by modulating the activity of adenylate cyclase enzyme thus affecting the intracellular cAMP levels [296]. Among these receptors, A2A and A2B receptor pathways activation by eADO are mainly involved in immunosuppression and, thus is presently considered as a main barrier to the immune therapy or tumor cells resistance to immune therapy. Adenosine pathway activation affects lymphocyte activation including tumor-infiltrating immune cells, myeloid cells, and tumor-associated stromal cells, and tumor cells consequently helping cancer cells to evade from anti-tumor immune cell response which results in therapy resistance [297]. So targeting CD39, CD73, and adenosine receptors in the presence of immune checkpoint therapy (anti-PDL1/PD1; ANTI CTLA-4) can prove to be a novel immunotherapeutic strategy against immune-resistant cancer cells. For example, combined treatment of Polyoxotungstate-1 (POM1; CD39 inhibitor) and anti-PD1 and CTLA-4 antibodies in B16-F10 (melanoma cells) transplanted mice model showed a remarkable reduction in tumor burden and increase in the survival of tumor-bearing mice [298]. Although rodent data is very promising but the key issue of this finding is wheather this can be translated in humans.

### Cancer cell drug Metabolism and resistance

Metabolism of drugs takes place through two different consecutive phases named “phase I” and “phase II”. Cytochrome P450s (CYPs) are major Phase I enzymes while glutathione-S-transferases (GSTs), and UDP-glucuronosyltransferases (UGTs), are the major enzymes belonging to Phase II enzymes and have a role in effecting anti-cancer drugs. The detailed mechanism of cancer cell metabolism in drug resistance is discussed below.

Many tumors have developed resistance against chemotherapy drugs, either through drug inactivation or by reducing action form. A major part of drug activation and inactivation takes place through the liver cytochrome P450 (CYPs) system [299, 300]. For example, the anticancer prodrug cyclophosphamide (translational inhibitor) used in a variety of human malignancies (breast, lymphoma) is first partially metabolized into active metabolite 4-hydroxy-cyclophosphamide (OHCP) by CYP isoforms [301]. OHCP forms an equilibrium with its tautomeric aldehyde “aldophosphamide” (ALDO), which is released and enters into other cancer/cells where it is enzymatically (3’-5’ phosphodiesterase) converted into DNA-RNA cross-linker phosphoramide mustard (PM) of clinical significance [302, 303]. It has been found that various resistant cancer cells (like Breast cancer etc.) have mutated CYP genes which reduces its drug-activating efficacy and leads to the breakdown or excretion of drugs without affecting cancer cells [299, 304]. So in such cases, CYP activation through gene therapy or using CYP mimetics can be very useful to restore the drug sensitivity of these cells.

Inside the body, the interactions between drugs and various types of proteins (like enzymes) activate drugs for their action [305]. However, cancer cells exhibit resistance to drugs by altering a catalytic site or structure of an enzyme which affects their interaction with drugs and thus culminates in their mode of action. For example, cytarabine (cytidine analog) resistant AML cells express mutated deoxycytidine kinase (cytarabine activator), reduce the activity of the drug and thus develop resistance [306, 307]. Similarly, doxorubicin (active) resistance in prostate and breast cancer is mainly due to enzymatic transformation into doxorubicinol (inactive) by the overexpressed enzyme Aldo–keto reductase, combination therapy is shown to be very effective in increasing therapeutic activity of DOX [308, 309].

Glutathione (GSH) which mainly catalyzes phase II of drug metabolism is a low molecular weight thiol molecule synthesized in cells. GSH plays an essential role in shielding cells from the toxicity of xenobiotic electrophiles, oxidative damage, and maintaining redox homeostasis [53]. GSH is reported to inhibit cisplatin-mediated cytotoxicity and activate ABC transporter activity thus inducing resistance in many cancers (L1210 leukemia cells etc.) against cisplatin [310, 311]. This opens a new treatment window of targeting GSH (like buthionine-sulfoximine) that might increase the sensitivity of resistant cancer cells toward cisplatin.

### Drug target alteration

The efficacy of a drug is influenced by its molecular target and alteration in the target decreases the impact of the drug's interaction with it. For example, Doxorubicin specifically targets topoisomerase II and prevents DNA replication. Nevertheless, some cancer cells in retaliation express mutated topoisomerase II with less binding potential to doxorubicin and thus become less sensitive to it [312]. Moreover, In non-small cell lung cancer (NSCLC), a mutation in the EGFR kinase domain (T790M) renders it resistant to gefitinib[313, 314]. Thus the secondary level of tyrosine kinase inhibitors such as ponatinib has been shown to have promising outcomes in such cases.

Cancer cells show drug resistance either by reducing uptake or enhancing the efflux of drugs or both through mutated receptors and transporters [315]. Membrane-bound transporters called organic anion-transporting polypeptides (like OATP1B1, OATP1B3, and OATP1A2) can transport paclitaxel, methotrexate, flavopiridol, tyrosine kinase inhibitors, irinotecan, cisplatin and plays a crucial role in the resistance in polyps of the large intestine and colon cancer [316–319]. Moreover in hepatocellular carcinoma (HCC), cholangiocarcinoma (CGC), and Chronic myeloid leukemia (CML) a lesser accumulation of anticancer drugs (like imatinib) seems to be mediated by a decrease in OATP1B1, OATP1B3, and OATP1A2 expression or their function [317, 320, 321]. Various studies have shown that organic cation transporter-1 (OCT1) is involved in the uptake of potent cationic anti-tumor drugs, such as Cisplatin, anthracyclines, and sorafenib and its activity is dysregulated in many cancers such as colon and liver cancer [322–325]. In addition, it has been demonstrated that imatinib uptake in CML is dependent on OCT1 expression and the degree of OCT1 expression is considered a useful biomarker for predicting the efficacy of imatinib-based therapy in leukemia patients [326]. The high-affinity copper transporter (CTR1) has been recently shown to transport platinum drugs, emphasizing the crucial function of CTR1 in platinum-drug sensitivity in cancer chemotherapy [327, 328]. A promising phase I clinical trial using cotreatment of trientine (a copper chelator) and carboplatin has shown CTR1-mediated higher cisplatin uptake and better outcomes [329, 330].

Most studied drug efflux-related genes are members of the ABC (ATP-binding cassette) superfamily [331]. It has been shown that isoforms of ABC transporter-like ABCB, ABCC, and ABCG families, are overexpressed in tumor cells, and are involved in chemotherapy resistance [331]. For example, higher expression of ABCB1 results in resistance to widely used anti-cancer Aurora Kinase Inhibitor (GSK-1070916) in colon cancer cells [332]. ABCC2 is considered to play a significant role in the resistance of colon cancer to platinum derivatives since it can effectively export glutathione-cisplatin conjugates, and in colon cancer cells, expression of multidrug resistance protein (MRP2 or ABCC2) was found significantly higher following cisplatin treatment [333, 334]. Nevertheless, non-ABC transporters are also reported to contribute to drug efflux and resistance. For instance, the copper-transporting P-type ATPases “Menkes and Wilson” proteins participate in clearing various intracellular drugs like cisplatin [335–337]. The major vault protein (MVP), or lung resistance-related protein (LRP), although not a pump, plays a similar role in transportation-mediated chemoresistance. MVP creates cytoplasmic nano-organelles called vaults that can enclose anticancer drugs like doxorubicin, and cisplatin (like in ovarian malignancies) and thereby lower their active intracellular concentrations thus mediate resistance [338–340].

## Autophagy and ER stress (UPR) are utilized by cancer cells to gain support for drug resistance

Autophagy is an intracellular catabolic process and is busy supporting cellular survival in various stressful conditions, however, in extreme cases, it is responsible for programmed cell death type 2 [133, 341]. In various types of cancer, autophagy can play a contrasting role in either supporting or inducing death and is context-dependent [342–347]. However, the role of autophagy in drug resistance is an emerging topic and a deep understanding of this relationship can prove to be therapeutically very crucial to curb various cancer types. Various studies have shown that autophagy plays a central role in drug resistance by recycling biomolecules, degrading deformed proteins and organelles, and thus preventing DNA damage [348–350]. Some reports have suggested that DNA damage response can also activate autophagy via ataxia telangiectasia mutated (ATM) and homologous recombination (HR) repair pathway [351, 352]. It has been found that an anthracycline drug epirubicin-mediated autophagy upregulates P-gp proteins and downregulates the NF-κB signaling pathway thus hindering activation of apoptosis and promoting drug resistance [353]. Tamoxifen sensitivity was enhanced in resistant estrogen receptor-positive breast cancer cells by autophagy inhibition, thus inducing cell death [354, 355]. In gastrointestinal stromal tumors, cotreatment of imatinib and autophagy inhibitors (like chloroquine) cause apoptosis [356, 357].The endoplasmic reticulum (ER) is an essential subcellular structure that maintains cellular homeostasis and can be disrupted by a variety of pathological conditions like cancer, resulting in induction of ER stress which if remains sustained can either kill cancer cells by induction of apoptosis and ferroptosis  or  help them to grow, survive and induce drug resistance if moderately activated [358]. ER stress induces UPR ,which is regulated by inositol-requiring enzyme-1 (IRE1α), activating transcription factor 6 (ATF6),  protein kinase RNA-like ER kinase (PERK), and such signaling pathways have been found to be overexpressed in various human tumors like breast, brain, liver, lung and pancreatic cancer cells contributing to their survival and drug resistance [358]. Hepatocellular carcinoma (HCC) is a deadly cancer worldwide and is commonly diagnosed in advanced stage and has high intrinsic drug resistance resulting in limited therapeutic efficacy. ER stress-mediated UPR via these UPR signaling pathways  including ATF6, IRE1, and PERK, was found to play a crucial role in the induction of HCC chemoresistance  by overexpressing ABC transporters such as MDR1, MDRP1, and MDRP2 [359, 360]. Moderate ER stress induction is correlated to the activation of the pro-survival genes that regulates amino acid metabolism, ER chaperones, redox reaction, protein folding, and autophagy [361].   In some cases..of cancer like in HCC, it has been found that endoplasmic reticulum stress-mediated activation of autophagy also plays a role in upregulating MRP1 that enhances intracellular drug or toxic heavy metal efflux thus protecting cells from apoptosis [362, 363]. Autophagy is shown to influence some crucial drug-resistant enzymes (detoxifying enzymes) like aldehyde dehydrogenase (ALD1A3) and thus arbitrate in acquired drug resistance in temozolomide-treated human glioblastoma cells [141]. In human ovarian cells (Polycystic ovarian syndrome- PCOS) aberrant autophagy induction upon release of the high mobility group box 1(HMGB1) plays a role in achieving insulin resistance by downregulating IRS-1, AKT, and GLUT4 translocation [364]. While in hepatocellular carcinoma cells, HMGB1 is shown to promote doxorubicin resistance by inducing AMPK-autophagy and protecting them from programmed cell death type 1 [365]. Autophagy is found to play a cytoprotective role in TNF-TRAIL death-resistant cells by sequestering, degrading, and dysfunctioning of caspase 8 protecting cancer cells to undergo death [366–368]. However, there are ample reports that prolonged or sustained autophagy activation can result in programmed cell death type 2. For example, resveratrol (plant-derived phytoalexin) treatment triggers a strong signal for autophagy (p62 accumulation) through JNK activation leading to death in imatinib-resistant chronic myelogenic leukemia cells (CML) [369, 370]. In some cases, autophagy activation supresses drug resistance and induces therapeutic dependent or independent death. For example, co-treatment of ABT-88 (polymerase protein inhibitor) and temozolomide has been shown to sensitize temozolomide-resistant glioma cells by inducing double DNA strand breaks and coincidently coactivating lethal autophagy and apoptosis [371]. So autophagy in most cases opposes the anti-cancer drug action of culminating cells by apoptosis, thus acting as a defensive cellular pathway and supporting drug resistance.

Targeting autophagy by using pharmacological inhibitors or by gene silencing in the presence of anticancer drugs can increase therapeutic efficiency and reduce drug resistance which can prove beneficial to increase the patient’s survival.

## Proteomic alteration in response to known chemotherapeutic drugs to achieve drug resistance (Fig. 6)

**Fig. 6:**
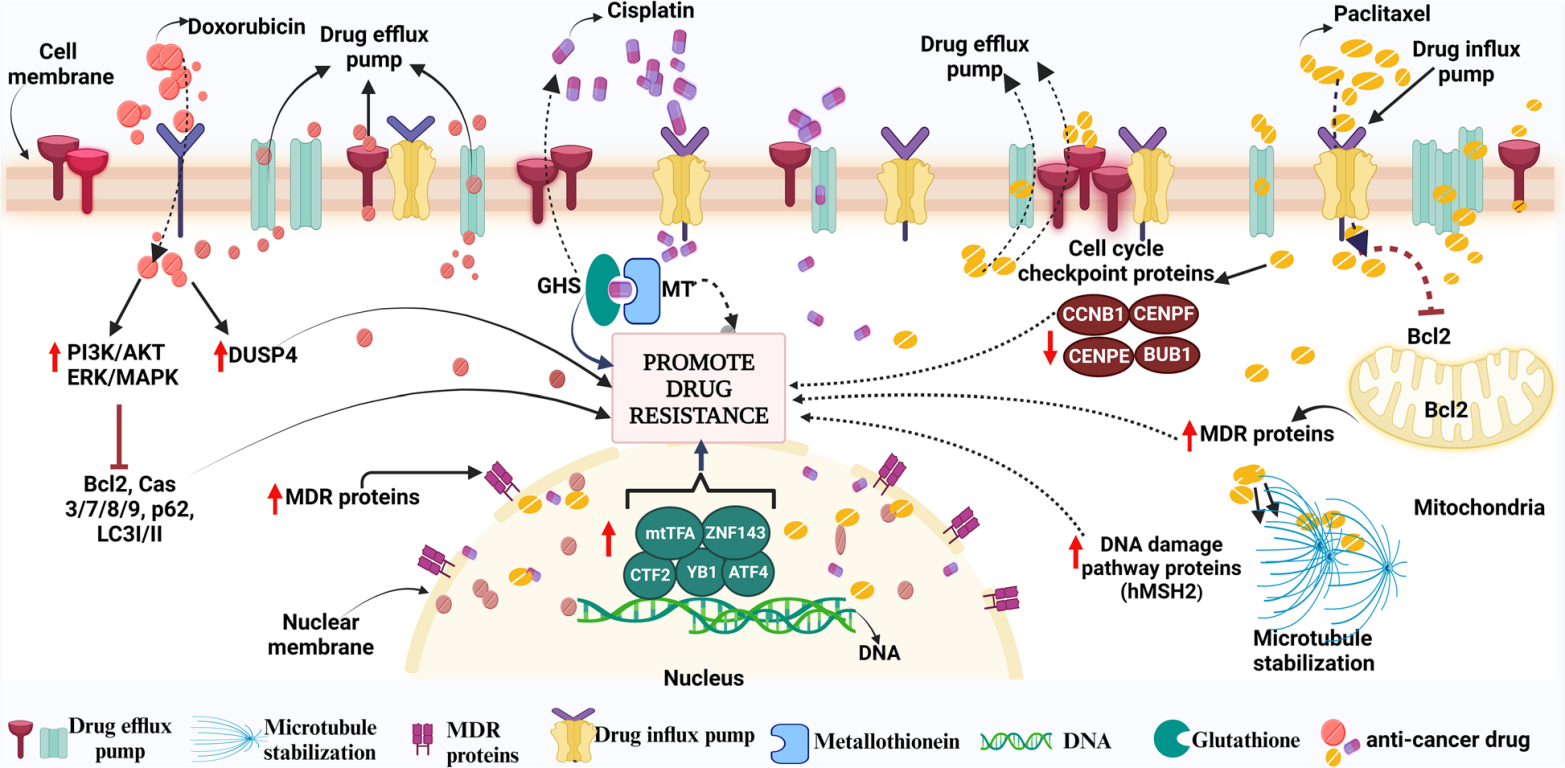
Depicting the resistance mechanisms of the different drugs at the proteomic level. Figure created with BioRender.com

Various known anti-cancer drugs in the market are mostly used against various malignancies with the positive outcome however some of the cancer cells alter their protein expression or morphology to become irresponsive and achieve resistance. Doxorubicin (DOX) is a well-known anti-cancer anthracycline drug that has been demonstrated to overexpress (off-target) signaling cascades PI3K/AKT, NF-κB and ERK/MAPK mainly responsible for its resistance by inhibiting apoptotic and autophagic death-related proteins- Bcl-2, caspase-3/7/8/9, p62, and LC3-I/II in the uterine and breast carcinoma [372–376]. Besides, doxorubicin resistance in numerous malignancies (like GC cells) has also been linked to the upregulation of dual specificity phosphatase 4 (DUSP4), or MAPK phosphatase 2. The cells became more sensitive to DOX when DUSP4 was knocked down [377]. Multidrug resistance transporters also contribute to DOX deprivation in cancers including bladder cancer, esophageal squamous cell carcinoma, or breast cancer, by overexpression of MRP2 (cMOAT or ABCC2) and are mainly responsible for its efflux [378–380]. TNBCs use different tactics against doxorubicin and defend themselves by upregulating the complement component system/cascade (C1ra, C1s, C2, C3, C4a, C5, C7, C8a, C8b) and activating MDSCs thus becoming immune resistant [381].

Likewise, resistance to cisplatin remains a barrier in the treatment of various cancers [382]. Upon entering the cell, it reacts with a range of molecules apart from DNA, such as sulfur-containing glutathione (GSH)/metallothionein (MT) which trap cisplatin and then eliminate it from the cell[383]. Moreover, Zinc-finger factor 143 (ZNF143), Y-box binding protein-1 (YB-1), activating transcription factor 4 (ATF4), CCAAT-binding transcription factor 2 (CTF2), DNA repair proteins (e.g., the product of the XRCC1 gene; YB1; etc.) and mitochondrial transcription factor A (mtTFA) are a few transcription factors that have been linked to CDDP resistance [384–386]. Another well-known drug is paclitaxel (PTX), which belongs to the taxane class of anticancer agents that affect the normal stability of microtubules during cell division and is effective on various cancers like breast, ovarian, etc. Although tubulin is the primary target for PTX, it has additionally been discovered to attack the mitochondria and block the activity of the apoptotic inhibitor protein Bcl-2 (B-cell Leukemia 2) [387]. Like other antineoplastic drugs, PTX treatment can induce resistance by inducing overexpression of the motor protein MCAK (leads to tubulin depolymerization), affecting membrane lipids (fatty acid synthase, Lipin, etc.), modified cell cycle checkpoint proteins (BUB1, CCNB1, CENPE, CENPF), enhanced DNA damage repair pathway proteins (like hMSH2), elevated efflux proteins (MDR1, MDR3, etc.[388–393].

## Discussion

Drug resistance is a huge clinical challenge that allows uncontrolled cancer progression and tumor relapse leading to reduced patient survival. Cancer cells achieve drug resistance under therapeutic pressure by modulating the tumor microenvironment, altering drug targets, and rewiring genetic, epigenetic and metabolic processes that help them to grow and survive under such conditions [394]. So it is vital to understand the stepwise drug-resistant mechanisms gained by different cancer cells to attain resistance towards particular chemotherapeutic drugs. Cancer cells within tumors are heterogeneous with many types of genetically altered cells like CSCs and behave differently to chemotherapeutic drugs [395]. Tumor heterogeneity has been shown to play a critical role in cancer drug resistance, by transforming a non-supportive, anti-tumorigenic environment into a supportive, pro-tumorigenic environment [15, 16]. Moreover, some of the tumor cells can induce the reprogramming of stromal cells and immune cells, inducing the secretion of diverse factors like cytokines, that enhance tumor progression and suppress cell death [396]. Besides, CSCs have emerged as key players in the intricate landscape of cancer drug resistance [397]. They acquire remarkable abilities to resist conventional treatments, driving disease recurrence and metastasis as has been detailed explained in Section-4. "CSCs play a key role in developing drug resistance and tumor relapse". CSCs unique properties, such as self-renewal and differentiation potential, upregulated surface membrane immune inhibitory ligands and release of various chemo/cytokines collectively contribute to therapy resistance and pose significant challenges in achieving long-term remission [398, 399]. Therefore targeting CSCs opens possibilities for innovative therapeutic strategies that can be used in combination with another chemotherapeutic drug that can hold promise in overcoming drug resistance and potentially improve patient outcomes. One of the prevailing mechanisms of drug resistance that needs to be focused on, is the involvement in the expulsion of hydrophobic drugs, facilitated by ATP-dependent ABC transporters [400]. A well-studied member of the ABC transporter, P-gp, an integral membrane protein, is frequently upregulated in diverse malignancies [400]. Specifically, gaining a comprehensive understanding of intricate mechanisms underlying multidrug resistance (MDR) in cancer cells is likely to play a pivotal role in the development of innovative approaches to cancer therapy in the coming years. However, more work is needed to be done from root level using high throughput assays (single cell level) of different sections of the same tumor samples to find a specific target/s for therapy or combination therapy as discussed above. Achieving this goal might address the evolving aggressive and resistant cancer cells leading to reduced severity and improving the survival of patients.

## Data Availability

Not applicable.

## Abbreviations

CSCs: Cancer stem cells
CTLA-4: Cytotoxic T lymphocyte-associated antigen 4
PD-1: Programmed Cell Death Protein 1
PD-L1: Programmed Death Ligand 1
NSCLC: Non-small-cell lung cancer
MDR: Multi-drug resistant
HCC: Hepatocellular carcinoma
DTP: Drug-tolerant persister
DNMTs: DNA Methyltransferases
DNAm: DNA methylation
5-FU: 5-Fluorouracil
P-gp: P-glycoprotein
Bcl-2: B-cell lymphoma 2
Bcl-xl: B-cell lymphoma-extra large
IAPs: Inhibitors of apoptosis
Bid: BH3 interacting-domain death agonist
Bax: Bcl-2 Associated X-protein
Bim: Bcl-2 Interacting Mediator of cell death
PUMA: P53 upregulated modulator of apoptosis
CRC: Colorectal carcinoma
DOX: Doxorubicin
MAPK: Mitogen-activated protein kinase
KMTs: Histone lysine methyltransferase
PRMTs: Protein arginine methyl transferase
GSH: Glutathione
DNA-PK: DNA-dependent protein kinase
SASP: Senescence-associated secretory phenotype
AML: Acute myeloid leukemia
Notch 1: Neurogenic locus notch homolog protein 1
Wnt: Wingless-related integration site
TGF-β: Transforming growth factor- β
ESCs: Embryonic stem cells
SOX2: Sex determining region Y-box 2
OCT4: Octamer-binding transcription factor 4
KLF4: Kruppel-like factor 4
SALL4: Sal-like protein 4
FOXM1: Forkhead box protein M1
Hh: Hedgehog
Chk: Checkpoint kinase
UBC: Urothelial bladder cancer
EMT: Epithelial-to-mesenchymal transition
MET: Mesenchymal-to-epithelial transition
TNBC: Triple-negative breast cancer
ABCG: ATP binding cassette subfamily G
FBXO21: F-Box Protein 21
GSTp: Glutathione S-transferase p
uPA: Urokinase Plasminogen Activator
HIF-1α: Hypoxia Inducible factor-1 α
CRISPR/Cas9: Clustered regularly interspaced short palindromic repeats/ Caspase 9
ATG: Autophagy-related
ROS: Reactive oxygen species
ALDH: Aldehyde dehydrogenase
TNBCSC: Triple-negative breast cancer stem cells
JAK/STAT: Janus Kinase/signal transducers and activators of transcription
IL: Interleukin
CCL2: CC-Chemokine ligand 2
CSF1: Colony stimulating factor 1
CSF2: Colony stimulating factor 2
HGF: Hepatocyte growth factor
MIF: Macrophage migration inhibitory factor
CX3CL1: C-X3-C motif Chemokine ligand 1
PGE2: Prostaglandin E2
SDF-1: Stromal cell-derived factor 1
LOX: Lysyl oxidase
CCL3: CC-Chemokine ligand 3
CCL5: CC Chemokine ligand 5
VEGF-A: Vascular endothelial growth factor A
PTN: Pleiotrophin
HMGB1: High-mobility group box 1
TAMs: Tumor-associated macrophages
EGFR: Epidermal growth factor receptor
IDO1: Indoleamine 2,3-dioxygenase 1
PGE: Prostaglandin
ENTPD2: Ectonucleoside triphosphate diphosphohydrolase 2
CD: Clusters of differentiation
MDSC: Myeloid-derived suppressor cells
CAR T cell: Chimeric antigen receptor T cell
CRS: Cytokine release syndrome
ZEB1: Zinc finger E-box binding homeobox 1
HDAC: Histone deacetylase
ATM: Ataxia-telangiectasia mutated
PCAF: p300/CBP-associated factor
FBXW7: F-box and WD repeat domain-containing 7
TWIST1: Twist-related protein 1
OXPHOS: Oxidative phosphorylation
LMP: Lysosomal membrane permeability
UCP2: Uncoupler protein 2
mTOR: Mammalian target of rapamycin
NF-κB: Nuclear factor- κB
STAT3: Signal transducer and activator of transcription3
MRP: Multidrug resistance protein
TRAIL: Tumor related apoptosis-inducing ligand
GBM: Glioblastoma multiforme
VIM: Vimentin
VDAC: Voltage-dependent anion channel
OHCP: 4-Hydroxy-cyclophosphamide
OATPs: Organic anion-transporting polypeptides
LRP: Lung resistance-related protein
UPR: Unfolded protein response
LC3: Light chain 3
CML: Chronic myelogenic leukemia cells (CML)
JNK: Jun N-terminal kinase
DUSP4: Dual specificity protein phosphatase 4
ERK: Extracellular signal-regulated kinase
ATF4: Activating transcription factor 4
ZNF143: Zinc-finger factor 143
YB-1: Y-box binding protein-1
BUB1: Budding uninhibited by benzimidazoles 1
CCNB1: Cyclin B1
CENPE: Centromere protein E
CENPF: Centromere protein F
